# Grand Challenges for Artificial Intelligence in Molecular Medicine

**DOI:** 10.3389/fmmed.2021.734659

**Published:** 2021-07-22

**Authors:** Frank Emmert-Streib

**Affiliations:** ^1^ Predictive Society and Data Analytics Lab, Faculty of Information Technolgy and Communication Sciences, Tampere University, Tampere, Finland; ^2^ Institute of Biosciences and Medical Technology, Tampere, Finland

**Keywords:** artificial intelligence, molecular medicine, machine learning, biomedicine, omics, data science, health data science, biomedical data science

## 1 Introduction

In recent years, novel methods from artificial intelligence (AI) and machine learning (ML) commonly referred to as data science (DS) enabled many advances in data-driven fields including computational biology, bioinformatics, network medicine, precision medicine and systems medicine (He et al., 2019; Rajkomar et al., 2019; Zou et al., 2019). Given the continuation of technological innovations that will further lead to new high-throughput measurements on all molecular levels, it can be expected that the importance of AI and ML for medicine and biomedicine will even increase in the future (Obermeyer and Emanuel, 2016; Emmert-Streib, 2021). For this reason, a scientific forum is needed for nurturing methodological developments and practical applications of AI, ML and general DS in molecular medicine allowing the community to disseminate and discuss recent results.

The Bioinformatics and AI Specialty Section aims to provide such a forum for publishing articles about the analysis of all types of Omics, clinical and health data for enhancing our understanding of molecular medicine. The emphasize is on either the application or the development of data-driven methods for diagnostic, prognostic, predictive or exploratory studies based on methods from AI or ML.

**FIGURE 1:**
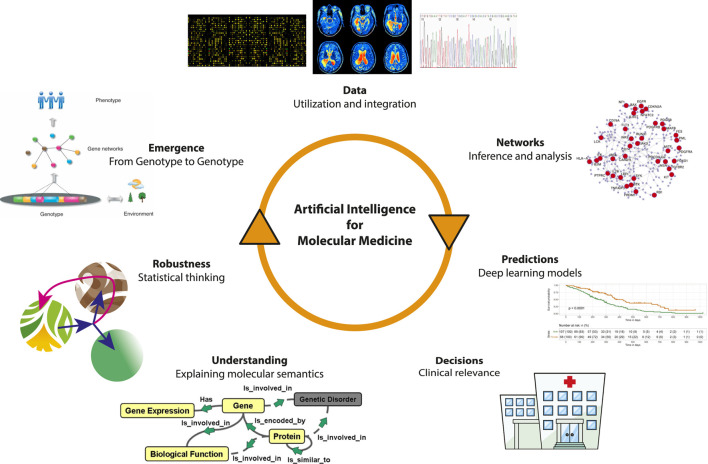
The iterative process of scientific discovery utilizing artificial intelligence and machine learning to enhance our knowledge about molecular medicine.

In Figure 1, we show an overview of the iterative process of scientific discovery utilizing artificial intelligence and machine learning to enhance our knowledge about molecular medicine. In the following, we discuss several of these topics that are in our opinion of particular relevance for the development of AI and ML in molecular medicine.

## 2 Data: Utilization and Integration

In (Feldman et al., 2012), “data” have been called “fuel” because it is like oil for scientific discoveries. For this reason, it is not surprising that we start by discussing the importance of data for molecular medicine. In general, all methods from data science, regardless if they have their origin in artificial intelligence, machine learning or statistics, are based on data (Emmert-Streib and Dehmer, 2019). In other words, a method alone is not capable of contributing anything of meaning for molecular medicine but the combination with data is required.

Nowadays there are many big data resources available that can be utilized for developing and testing methods. Prominent examples thereof are The Cancer Genome Atlas Research Network (TCGA) (The Cancer Genome Atlas Research Network, 2008), Gene ontology (GO) (Ashburner et al., 2000), Gene Expression Omnibus (GEO) (Edgar et al., 2002) or Library of Integrated Network-based Cellular Signatures (LINCS) (Koleti et al., 2017). Interestingly, the idea that such data can also be used for making novel discoveries about the molecular understanding of disorders is so far largely underexplored.

A common problem encountered is how the diverse and often heterogeneous data can be integrated in a meaningful and sound way (Zitnik et al., 2019). Traditionally, one tried to accomplish this by the normalization of data with the hope that this allows the pooling of data, i.e., two or more data sets can be combined, from different sources. While this approach is applicable in certain situations it does not offer a generic solution. Instead, a conceptual approach that could be of great practical relevance in this context is provided by transfer learning (Pan and Yang, 2009). The basic idea of transfer learning is to utilize data from two different domains and to use both for learning a so called target task. Importantly, the underlying feature spaces of both domains can be different. Hence, this framework allows to utilize data from different domains without actually combining them. For instance, data from DNA microarrays can be used to improve tasks for RNA-seq data or even to utilize imaging data, e.g., from X-Rays or fMRIs, or text data from electronic health records (eHR) for the same target task. Other machine learning paradigms that could be of relevance are multi-task learning or semi-supervised learning (Chapelle et al., 2006; Zhang and Yang, 2018).

## 3 Networks: Inference and Analysis

Another type of approach that is of crucial relevance for molecular medicine is network-based approaches (Vidal, 2009). Specifically, there have been many studies inferring various types of gene regulatory networks (GRNs), including transcription regulation networks, protein interaction networks, metabolic networks or signalling networks (Emmert-Streib and Dehmer, 2018). Each of these provide useful information about molecular interactions on the cellular level (Emmert-Streib et al., 2014). However, in order to obtain a full systems biology understanding an integration of such networks is needed. Hence, multi-scale network studies are needed to provide us with comprehensive blue-prints about the hierarchical molecular organization pattern (Yu and Gerstein, 2006; Ravasz, 2009).

A field that is dedicated for utilizing such approaches is network medicine (Barabási, 2007). A particular example for the utility of networks is to study the relations between disorders and genes (Goh et al., 2007; Emmert-Streib et al., 2013). Importantly, instead of focusing on individual disorders or genes at a time, network medicine aims at providing insights into the intricate interrelations among all such entities. This allows not only the exploitation of common biological processes or pathways but also to make predictions, e.g., about the drug repurposing (AY et al., 2007; Pushpakom et al., 2019). Hence, networks provide efficient means for studying basic molecular biological questions of disorders and pharmacogenomic problems to gain insights into treatment options for patients.

## 4 Predictions: Deep Learning Models

A good example to show that machine learning and artificial intelligence are dynamical fields with constant innovations is deep learning (LeCun et al., 2015). Methods of this type came to the awareness of the general community around 2012 and have since then contributed to enhance our understanding in many domains. One particular reason contributing to the success of deep learning methods is the flexibility they offer for building neural networks of different tasks. As a result, nowadays a large number of network architectures is known, e.g., Convolutional Neural Networks (CNN), Long Short-Term Memory networks (LSTM) or Deep Belief Networks, that have been applied in a large variety of application domains (Schmidhuber, 2015; Emmert-Streib et al., 2020a).

One particular deep learning model for the analysis of text data that received considerable attention is BERT (Bidirectional Encoder Representations from Transformers) (Devlin et al., 2019). BERT is an autoencoding language model trained using stacked encoder blocks from transformers with a masked language modeling (MLM) to learn word-embeddings bidirectionally. Part of the success of this model is its flexibility to be utilized for a number of different prediction tasks, including named entity recognition, question answering and relation detection (Perera et al., 2020). Hence, this model is of great relevance for analyzing, e.g., electronic Health Records (eHR) from hospitals (Lee et al., 2019; Li et al., 2019).

## 5 Decisions: Clinical Relevance

Of particular practical relevance for molecular medicine are studies investigating diagnostic, predictive, prognostic or therapeutic signatures of biomarkers. The reason for this it that such studies have the potential to inform clinical decision making by influencing the diagnosis or treatment of patients in profound ways. The surge of genomics data provides ample opportunities for such studies and one key issue of these is feature selection. Specifically, while the number of molecular entities, e.g., about genes or mRNAs, is in the tens of thousands, interpretable models aim to limit this number to the smallest possible number.

Another interesting topic in this context is the utility of network biomarkers. In contrast to traditional approaches that are based on, e.g., sets of genes or proteins, network biomarkers utilize structural features from gene regulatory networks (Chen et al., 2012; Zeng et al., 2013). This converts a structureless set of genes (sometimes called gene bag) into a complex entity conveying more predictive and interpretable information. As a side-note we would like to mention that this could be also beneficial for the visualization of results and the doctor-patient communication in order to explain therapeutic measures.

## 6 Understanding: Explaining Molecular Semantics

A common goal of all above approaches is to enhance our understanding of the molecular bases of disorders. In order to see that this is a non-trivial endeavour let’s discuss some examples. Deep learning models have been criticized for being black-box models (Adadi and Berrada, 2018). That means such models are good for making predictions but defy a straight-forward interpretation making the models non-explainable (Emmert-Streib et al., 2020b). This is particularly problematic in a medical context involving humans because this utimately means that clinical decisions, e.g., based on the analysis of personal genomics data, cannot be explained to the patient.

Another example is given by biomarkers. In general, biomarkers are used for diagnostic, prognostic, predictive or therapeutic purposes to make decisions about the care of a patient (Califf, 2018). It is widely believed that aside from this clinical utility based on the predictive capabilities of such signatures, biomarkers are also offering insights into the molecular functioning of biological processes and their causal involvement in disorders (Van De Vijver et al., 2002; Cuzick et al., 2011). However, for prognostic signatures of breast cancer it has been demonstrated that this is not the case (Venet et al., 2011; Manjang et al., 2021). This implies that also the prognostic signatures are black-box models with sensible predictions of breast cancer outcome but no value for revealing causal connections. Hence, such models have a predictive utility, e.g., for applications in the clinical practice but no biological utility for enhancing our understanding of breast cancer biology. If similar results are observed for other cancer types or different disorders remains to be seen.

From these examples one can see that establishing a good prediction model does not impliy that we also obtain immediately an understanding of the molecular semantics offered by disorders. Hence, ideally, causal prediction models are required that provide prediction capabilities along with an interpretable structure for giving causal explanations of molecular activities (Holzinger et al., 2019). In case such ideal models are unachievable one needs measures for quantifying these deficiencies.

## 7 Robustness: Statistical Thinking

An aspect that does not receive enough appreciation is the fact that any type of the analysis of data from molecular medicine requires statistical considerations. That means even modern developments in AI and ML do not make a statistical understanding obsolete but are built upon it. This includes, for instance, ensuring the reproducability of studies (Peng, 2011; Begley and Ioannidis, 2015), multiple testing corrections of hypotheses (Noble, 2009) or the regularization of regression models (Tibshirani, 1996). Of particular interest are studies that clarify the understanding of problems of widely used methods or approaches (Ioannidis, 2005; Tripathi et al., 2013; Wasserstein and Lazar, 2016). Hence, investigations that enhance our understanding of molecular medicine by applying any form of *statistical thinking* are welcome to advance bioinformatics because only such approaches lead to the robustness of findings that are of biological and clinical significance (Vingron, 2001).

## 8 Emergence: From Genotype to Phenotype

Finally, we would like to emphasize that molecular medicine aims to study the connection between genotype and phenotype (Ginsburg and Willard, 2009; Collins and Varmus, 2015). That means, while aberrant molecular processes give rise to various forms of disorders, those molecular processes should not be studied in isolation but their phenotypic consequences need to be systematically documented. However, this requires to bridge from genotype to phenotype (Noble, 2008a; Gjuvsland et al., 2013; Ritchie et al., 2015). For practical approaches ’networks’ have been suggested to capture relevant information as an intermediate layer (Emmert-Streib and Glazko, 2011; Carter et al., 2013; Kim and Przytycka, 2013), however, further instigations are needed, e.g., to merge such approaches with predictive models.

On a theoretical note, we would like to highlight that the above problem is actually severe because it requires an understanding of *emergence* (Noble, 2008b; Pigliucci, 2010). Hence, reductionist approaches are prone to fail in molecular medicine which possesses major challenges for conceptual approaches provided by AI or ML to overcome such limitations (Mazzocchi, 2012). Hence, even personalized medicine or precision medicine depend on our theoretical understanding of the biological complexity of emergent features arising from the transition between the genotype to the phenotype.

## 9 Conclusion

The Bioinformatics and AI Specialty Section of Frontiers in Molecular Medicine will provide a venue for world-class interdisciplinary research addressing the above, and many more challenges arising in the future. In order to provide a forum for the exchange of ideas and growth of innovations for a multi-disciplinary research community, the journal does not only publish Original Research and Review articles but a number of additional paper types. For instance, the journal welcomes submissions for the following article types: Hypothesis and Theory, Perspective, Opinion and General Commentary. This will allow to express the perspectives and opinions of the community and to discuss recent developments critically. Furthermore, the journal publishes also Technology and Code articles which present 1) new software, 2) new applications of software or 3) implementations of existing algorithms under novel settings.
